# Impact of Host Cell Line Adaptation on Quasispecies Composition and Glycosylation of Influenza A Virus Hemagglutinin

**DOI:** 10.1371/journal.pone.0027989

**Published:** 2011-12-07

**Authors:** Jana Verena Roedig, Erdmann Rapp, Dirk Höper, Yvonne Genzel, Udo Reichl

**Affiliations:** 1 Max Planck Institute for Dynamics of Complex Technical Systems, Magdeburg, Germany; 2 Friedrich-Loeffler-Institut (FLI), Greifswald - Insel Riems, Germany; 3 Otto-von-Guericke-University, Chair of Bioprocess Engineering, Magdeburg, Germany; American University in Cairo, Egypt

## Abstract

The genome of influenza A viruses is constantly changing (genetic drift) resulting in small, gradual changes in viral proteins. Alterations within antibody recognition sites of the viral membrane glycoproteins hemagglutinin (HA) and neuraminidase (NA) result in an antigenetic drift, which requires the seasonal update of human influenza virus vaccines. Generally, virus adaptation is necessary to obtain sufficiently high virus yields in cell culture-derived vaccine manufacturing. In this study detailed HA *N*-glycosylation pattern analysis was combined with in-depth pyrosequencing analysis of the virus genomic RNA. Forward and backward adaptation from Madin-Darby Canine Kidney (MDCK) cells to African green monkey kidney (Vero) cells was investigated for two closely related influenza A virus PR/8/34 (*H1N1*) strains: from the National Institute for Biological Standards and Control (NIBSC) or the Robert Koch Institute (RKI). Furthermore, stability of HA *N*-glycosylation patterns over ten consecutive passages and different harvest time points is demonstrated. Adaptation to Vero cells finally allowed efficient influenza A virus replication in Vero cells. In contrast, during back-adaptation the virus replicated well from the very beginning. HA *N*-glycosylation patterns were cell line dependent and stabilized fast within one (NIBSC-derived virus) or two (RKI-derived virus) successive passages during adaptation processes. However, during adaptation new virus variants were detected. These variants carried “rescue” mutations on the genomic level within the HA stem region, which result in amino acid substitutions. These substitutions finally allowed sufficient virus replication in the new host system. According to adaptation pressure the composition of the virus populations varied. In Vero cells a selection for “rescue” variants was characteristic. After back-adaptation to MDCK cells some variants persisted at indifferent frequencies, others slowly diminished and even dropped below the detection limit.

## Introduction

Influenza A virus is a highly virulent human and animal pathogen, particularly due to its ability to cause severe disease. Hygiene measures and vaccination still represent the most efficient prevention strategies. Since several years cell culture-based processes are being established to overcome problems of traditional egg-based vaccine manufacturing such as egg supply shortages and difficulties in rapid scale-up during pandemics, as well as to increase manufacturing capacities. The need for the seasonal reformulation of influenza vaccines is attributed to the virus' ability to rapidly adapt to changing environments. Virus adaptation is one of the most important processes in virus evolution, and a crucial factor to be taken into account for seasonal and pandemic vaccine production. On the one hand, adaptation allows the virus to cross species boarders, evade immune or therapeutic pressures and optimize its replication in a given host system [1]. On the other hand, it challenges manufacturers to adapt emerging strains to existing egg-based or cell-culture-based system processes to obtain maximum yields for formulation of potent vaccines [2], [3]. Escape from immune pressure, balancing host cell receptor binding avidity of input virus with the release of progeny virus as well as adjustment to altered endosomal pH-values or to different, specific sialic acid containing host cell receptors have been described as driving forces for adaptation processes in virus evolution [2], [4]–[6]. During the initial step of infection the viral surface glycoprotein hemagglutinin (HA) binds to host-specific sialic acid receptors [7]. The following receptor-mediated endocytosis leads to a drop in pH-value, causing conformational changes of the HA subunits that enable the release of the viral RNA into the cytoplasm of the host cell [8]. Besides this key role in virus replication, HA is a highly abundant protein in the membrane of the virus particle, and it represents the major component in influenza vaccines due to its ability to induce a strong and protective immune response [9], [10].

Glycoproteins such as HA can be considered as a collection of different glycoforms or glycosylation variants [11]. A glycoprotein's characteristic is described by specific activity [12], antigenicity [12]–[14], binding avidity [15] and specificity [16]. It can be influenced significantly by changes, respectively, differences in *N*-glycosylation, reflecting variations in glycosylation site occupancy (macroheterogeneity) and in structure of sugar residues (microheterogeneity). This complexity on the glycomic level is increased additionally by a co-existence of related virus subpopulations on the genomic and therefore also on the proteomic level, so-called quasispecies [17]–[19]. On the one hand, new influenza variants can originate from the virus' ability to newly reassort (genetic shift) [20], which allows the generation of high-growth reassortants in vaccine manufacturing [21]. On the other hand, the high error rate of the viral polymerase raises constantly new variants [22], which only differ in single or few amino acid positions, resulting in variations of the virus genoms. Further, natural selection leads to the adaptation of a given virus as an evolutionary response to “new-host-pressure” [20]. The frequency of a virus variant in a population largely depends on its ability to survive and reproduce – i.e. its fitness [23]. However, if coupled to high fitness genotypes, low fitness virus variants can be maintained at higher levels than expected [24]. For vaccine manufacturing this implies the requirement of multiple variant selection steps for virus seed preparation. In general, human isolates replicate poorly in eggs. In order to minimize the risk of contamination with other human pathogens, clinical specimens of the strains recommended for the next season's vaccine formulation are usually blind-passaged in embryonated chicken eggs by WHO Collaborating Centers. Further process optimizations are performed by manufacturers. Here variants that replicate well are selected to be reassorted to high-yield laboratory viruses to generate virus seeds used in production. Due to possible antigenic drift during each of these steps antigen identity testing and sequence analyses are required [25], [26].

In this study we investigated the stability of the HA *N*-glycosylation pattern over 10 successive virus passages in Madin-Darby canine kidney (MDCK) cell culture. In addition, HA-stability was characterized for MDCK and Vero (African green monkey kidney) cells for up to 96 hours post infection (hpi) and 360 hpi, respectively. Furthermore, the impact of adaptation from MDCK to Vero cells and back on HA *N*-glycosylation and composition of the virus population was studied. Therefore, two closely related MDCK cell-adapted influenza A virus Puerto Rico/8/34 (*H1N1*) strains were adapted to growth on Vero cells. Resulting Vero cell-adapted viruses were subsequently back-adapted to replicate in MDCK cells. Finally, the consequences on HA level of different virus adaptation processes from MDCK cell to Vero cell based production and back were monitored. Therefore, detailed HA *N*-glycosylation pattern analysis of this major vaccine antigen was combined with in-depth pyrosequencing analysis of the HA encoding RNA segment 4. This allowed a characterization of quasispecies' composition.

## Materials and Methods

### Cell lines

Adherent MDCK (No. 84121903) or Vero (No. 88020401) cells were purchased from ECACC (Salisbury, UK). The cells were either grown in T75- and T175- flasks at 5% CO_2_ and 37°C or in roller bottles with closed caps at 37°C. For cell growth GMEM (Invitrogen/Gibco, #22100-093, Darmstadt, Germany) was supplemented with 5.5 g/L glucose (Roth, #X997.3, Karlsruhe, Germany), 2 g/L peptone (IDG, #MC33, Lancashire, UK), 10% FCS Invitrogen/Gibco, #10270-106, Darmstadt, Germany) and 4 mg/mL NaHCO_3_ (Roth, #6885.3, Karlsruhe, Germany).

### Virus

Infections were performed with influenza A/Puerto Rico/8/34 (*H1N1*) from either the National Institute for Biological Standards and Control (NIBSC, #06/114, South Mimms, UK) or the Robert Koch Institute (RKI, Amp. 3138, Berlin, Germany) according to Genzel et al. [27]. In the following these virus strains are referred to as PR/8/34 (NIBSC) or PR/8/34 (RKI), respectively. Briefly, for infection and virus production the serum-free growth medium was supplemented with a final concentration of 5 U/mL trypsin (Invitrogen/Gibco, #27250-018, Darmstadt, Germany), which was prepared in phosphate buffered saline (PBS) according to the activity given by the supplier.

### Virus growth for stability testing of HA N-glycosylation pattern and cell line adaptation studies

All infections were performed when cells were confluent using 0.2 mL virus seed for T75 flasks, 0.4 mL for T175 flasks or 1 mL for roller bottles (a volume-based passaging was chosen since the time required for determination of the number of infectious virus particles by TCID50 or plaque assays was too long for the experimental setup). Furthermore, Genzel et al. showed that the multiplicity of infection (moi) has no effect on final HA virus titers, only on the time needed to reach this maximum HA value [28]. To generate the initial virus inoculum (passage 1), MDCK cell-adapted virus seed was thawed and propagated for 24 h in MDCK cells using a moi of 2 or 0.03 for PR/8/34 (RKI) and (NIBSC), respectively. All successive passages (2 to 11) were infected with virus culture supernatant of the previous cultivation provided that HA-titers were above 1.8 log HA units/100 µL (1.8 HAU) - the minimal HA-titer required for *N*-glycan analysis. Overall, the initial MDCK cell-adapted virus was passaged five times in Vero cells and subsequently for back-adaptation up to five times in MDCK cells. As a control the virus seed was passaged in MDCK cells only (initial moi 0.3). In order to monitor the impact of harvest time points, MDCK (initial moi 0.3) and Vero (initial moi 0.1) cell-adapted virus seed was propagated in MDCK and Vero cells, respectively and isolated at different time points within the time frame of interest for MDCK as well as Vero cell-derived virus. All controls were performed in roller bottles using PR/8/34 (RKI).

### Hemagglutination assay

Influenza virus from cell culture supernatant was titrated by hemagglutination based on the method by Kalbfuss et al. [29]. Samples were serially diluted 1:2^0.5^ in PBS resulting in a final volume of 100 µL using round-bottomed 96-well plates. 1.9–2.1×10^6^ red blood chicken cells in a total volume of 100 µL in PBS were added and incubated for 3 h to 4 h at room temperature. Light extinction was measured at 700 nm and plotted over the logarithm of the inversed dilutions. The end point was defined as the point of inflection - the last dilution (d) showing complete hemagglutination - corresponding to 1 HAU (HAU  =  HA Units  =  -log_10_ d/100 µL). A sample's HA-titer is calculated by considering the dilution factor of the end point. Consequently, if e.g. the end point is reached at a dilution of 1/64 (1/10^1.8^), this corresponds to an HA-titer of 1.8 HAU for the undiluted sample.

### Virus purification, N-glycan analysis and data evaluation

**Figure 1:**
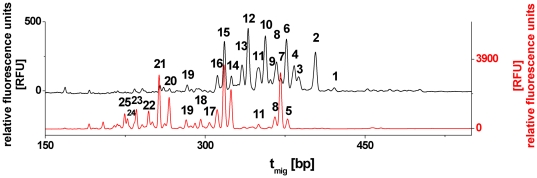
Peak annotation of MDCK and Vero cell-specific HA *N*-glycosylation patterns of influenza A/PR/8/34 (*H1N1*). Relative fluorescence units (RFU) plotted over normalized migration time in basepairs (bp). The shifted overlay of the normalized capillary electropherograms, represents MDCK cell-specific (black) and Vero cell-specific (red) glycosylation patterns.

Virus purification by gradient step centrifugation, sample preparation, *N*-glycan analysis and data evaluation were performed according to Schwarzer et al. [30], [31], but applying an optimized protocol. This included the substitution of 20 mM sodium hydrogen carbonate _(aq)_ with 50 mM ammonium hydrogen carbonate _(aq)_ during enzymatic in-gel-deglycosylation and *N*-glycan extraction as well as the reduction of the final sample volume for PNGaseF-digestion to 60 µL, which resulted in an increased enzyme concentration. Labeled samples were desalted by size exclusion chromatography as described by Schwarzer et al. [30]. Briefly, capillary gel electrophoresis with laser-induced fluorescence detection (CGE-LIF) was performed in an ABI PRISM 3100-Avant genetic analyzer (Applied Biosystems, Foster City, California, USA). The purified samples were diluted in HiDi formamide (#4311320C, Applied Biosystems, Foster City, California, USA). The *N*-glycan pool of an HA sample was separated by capillary gel electrophoresis, resulting in capillary electropherograms, which – after migration time normalization - corresponds to the *N*-glycosylation pattern, in which relative fluorescence units (RFU) are plotted over the normalized migration time (t_mig_) in base pairs (bp). In *N*-glycosylation patterns, one peak corresponds to at least one distinct *N*-glycan structure. The total peak height (TPH) is calculated as the sum of all peaks and is set to 100%. Differences in relative abundances are expressed in percentage of relative peak height (RPH). Accordingly, overlays of multiple *N*-glycosylation patterns allow a direct comparison of different HA *N*-glycan pools. In the following peaks are annotated as shown in figure 1. Data were processed as published previously [30]–[32].

### Pyrosequencing and sequence assembly

Segment 4 (HA) of influenza A was sequenced using the Genome Sequencer FLX instrument (Roche, Mannheim, Germany). For DNA preparation the protocol of Höper et al. [33] was applied with the modifications described by Leifer and colleagues [34]. RT-PCR was conducted as described by Höper et al. [35]. For reverse transcription of the RNA genome segment, the Transcriptor High Fidelity cDNA Synthesis Kit (Roche, Mannheim, Germany) and for amplification of the cDNA the iProof High-Fidelity Master Mix (Bio-Rad Laboratories GmbH, München, Germany) was used. The sequencing libraries were generated according to Wiley et al. [36] followed by DNA binding to library capture beads and the recovery of single-stranded template DNA (sstDNA) library. For bead-bound clonal amplification, the DNA libraries were subjected to duplicate emulsion PCRs (emPCR) with the GS emPCR kit I (Roche, Mannheim, Germany), according to the manufacturer's instructions (2 copies per bead). After bead recovery and enrichment, the beads were sequenced using a GS LR70 sequencing kit (Roche, Mannheim, Germany) and the appropriate instrument run protocol provided by the vendor. The resulting sequencing reads were sorted according to the genome segments and assembled into one contig (i.e., a set of overlapping sequencing reads) per segment using the GS FLX sequence assembly software newbler (version 2.3; Roche, Mannheim, Germany). During the assembly, primer sequences were trimmed-off the raw data. All sequences were deposited in the GISAID EpiFlu database (www.gisaid.org), the accession numbers are compiled in the supplementary (table S1). For quasispecies analysis, a mapping of the raw sequencing reads along the reference sequence was performed using the GS FLX reference mapper software (version 2.3; Roche, Mannheim, Germany) with the default parameters (also during the mapping primer sequences were trimmed off the raw data). Briefly, ”high confidence” differences were defined by a combination of flow signal, quality score and difference type information: generally, at least three non-duplicate reads with the difference are required. Besides, there must be both forward and reverse reads showing the difference, unless there are at least seven reads with quality scores over 20. If the difference is a single-base over- or undercall (e.g. CC instead of C, or A instead of AA), the reads with the difference must form the consensus of the sequenced reads (i.e. at that location the overall consensus must differ from the reference) and the signal distribution of the differing reads must vary from the matching reads and the number of bases in that homopolymer of the reference (GS FLX Software Manual 2.17.1.14).

In the following, amino acid substitutions during the adaptation processes will be annotated by the one letter amino acid code for the original residue (in passage 1), followed by the position number of the residue, followed by the one letter code of the substituting amino acid in the later passage. Insertions and deletions are indicated by a minus sign (-) at the first or last position, respectively.

### Sample storage

Virus containing cell culture supernatants as well as virus isolates were stored at −80°C; HA *N*-glycans for glycosylation pattern analysis were stored at −20°C. Sealed Coomassie-stained polyacrylamide gels with HA bands (#161-1155 or #161-1158, BioRad, Hercules, Canada) were stored at 4°C.

### Stability of HA N-glycosylation pattern over multiple virus passages and over different harvest time points

One essential condition for the characterization of adaptation processes of influenza viruses A/PR/8/34 (*H1N1*) to different host cell lines is the stability of the HA *N*-glycosylation pattern over multiple virus passages in one cell line. For MDCK cell-derived PR/8/34 (RKI), HA-titers of 10 successive virus passages ranged between 2 and 2.4 HAU at 24 hpi (data not shown). Furthermore, all HA *N*-glycosylation patterns feature the same 15 main peaks (no. 1-4, 6-16) throughout all passages (figure S1A). The standard deviations (SD) of RPHs range between 0.25% and 1.14% (figure S1B). Overall, these results demonstrate a high stability of the HA *N*-glycosylation pattern over 10 successive virus passages in the same host cell system.

Furthermore, previous experiments showed that for guaranteed *N*-glycan analysis samples with a minimum HAU-value of 1.8 are required (data not shown). With differences in replication dynamics during adaptation to a new cell line, i.e. slow replication dynamics during initial adaptation steps and fast progress of infection towards the end, different time points for sampling were required to achieve a minimum of 1.8 HAU. Hence, the impact of harvest time point on HA *N*-glycosylation patterns was investigated for Vero cell-adapted PR/8/34 (RKI) virus in Vero cells, and for MDCK cell-adapted virus in MDCK cells. For all sampling points (24, 96 hpi) during MDCK cell-based virus replication, the HA *N*-glycosylation pattern was dominated by the same 15 main peaks (no. 1 - 4, 6 - 16; figure S2A), exhibiting SD for RPH between 0% and 2.3% (figure S2B).

In contrast, the pattern from Vero cell-derived HA was defined by 16 main peaks (no. 5, 7, 8, 11, 15 - 25, figure S3A). Regarding the complete time span (sampling points 48, 96, 200, 360 hpi) the SD for RPH ranged from 0.1% to 6% (figure S3B). Considering only the time span until 96 hpi, SD comparable to the MDCK time series were observed (SD<2.7%). Taking into account the comparatively long sampling period of 360 h, the higher SD were most likely caused by *N*-glycan degradation or synthesis. In particular, the RPH of peaks 5 and 7 steadily increased (it almost doubled for peak 7) and the RPH for peaks 16, 20, 22 to 25 steadily decreased over time. However, even despite those changes of RPH, the HA *N*-glycosylation pattern itself was stable over 360 h.

Overall, results demonstrated a highly reproducible HA *N*-glycosylation pattern for both cell lines concerning the number of glycan structures. For Vero cells, but to a certain degree also for MDCK cells (due to smaller time frame), the relative amounts (RPH values) of single structures present in the glycan pool, were dependant on the harvest time point of the virus-containing supernatant.

## Results and Discussion

### Adaptation of PR/8/34 from MDCK to Vero cells and back

**Figure 2:**
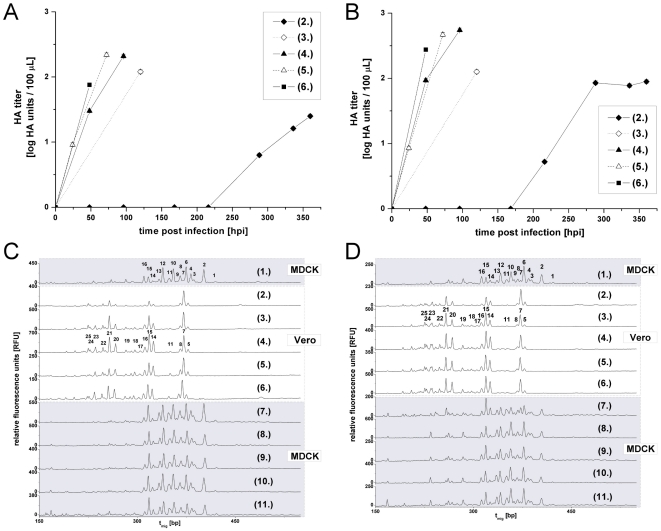
HA-titers and shifted overlays of HA *N*-glycosylation patterns during influenza virus A/PR/8/34 (*H1N1*) adaptation from MDCK to Vero cells. MDCK cell-adapted virus seed from RKI (A) as well as from NIBSC (B) replicate poorly in the first Vero cell passage (♦, passage 2). Passage (3) to (6) exhibit increased virus fitness (◊, ▴, *Δ*, ▪). For the HA-assay the 95% confidence interval is maximum 15%. (C, D) The normalized capillary electropherograms altogether represent 11 virus passages of the virus from RKI (C) and NIBSC (D): (1) virus seed, (2) to (6) adaptation to Vero cells, (7) to (11) back-adaptation to MDCK cells. MDCK and Vero cell-derived HA *N*-glycosylation patterns dominated by 15 peaks and 16 peaks, respectively.

In 2010 Genzel et al. showed that viruses often require adaptation to new host cells to optimize yields and time of harvest. In order to further characterize this adaptation process and to investigate possible biological mechanisms MDCK cell-adapted virus was propagated for 24 h in MDCK cells (passage 1). As a result HA-titers of 1.9 HAU and 2.2 HAU were obtained for the strains from RKI and NIBSC, respectively (data not shown). Supernatant of this passage served as the virus seed for a first infection of Vero cells (passage 2). For the RKI-strain virus-release was not detected before 288 hpi (0.8 HAU, figure 2A) and the maximal HA-titer of 1.4 HAU was finally reached 360 hpi. For the NIBSC-strain virus release was first detected 216 hpi and virus replication was continued until 360 hpi, exhibiting a final HA-titer of 1.95 HAU (figure 2B). An aliquot of these supernatants served as virus seeds for the next infections of Vero cells (passage 3) and so on (passage 4 to 6). During this virus adaptation to Vero cells, viral fitness of the RKI-strain improved: The time required to achieve specific HAU-values (≥ 1.4 HAU) decreased, whereas maximum HA-titers increased from passage 2 to 4. No significant differences were detected for passage 4 to 6, indicating the completion of the adaptation process (figure 2A). For the NIBSC-strain, the second passage in Vero cells (passage 3) reached 2.1 HAU at 120 hpi, and passage 4 reached 2.74 HAU at 96 hpi. For all subsequent passages the time required to achieve a HA-titer of at least 1.8 HAU as well as the maximal titers were more or less the same. This indicated the completion of the adaptation process (figure 2B). The following five virus passages (7 to 11) were again performed in MDCK cells to monitor virus back-adaptation. Here, for the RKI- as well as the NIBSC-strain all titers ranged between 2 HAU and 2.5 HAU and were reached between 48 hpi and 96 hpi (data not shown). In contrast to the adaptation to Vero cells, no impact on HA-titer level and virus release dynamics was observed during back-adaptation.

### Host cell-specificity of HA N-glycosylation patterns

**Figure 3:**
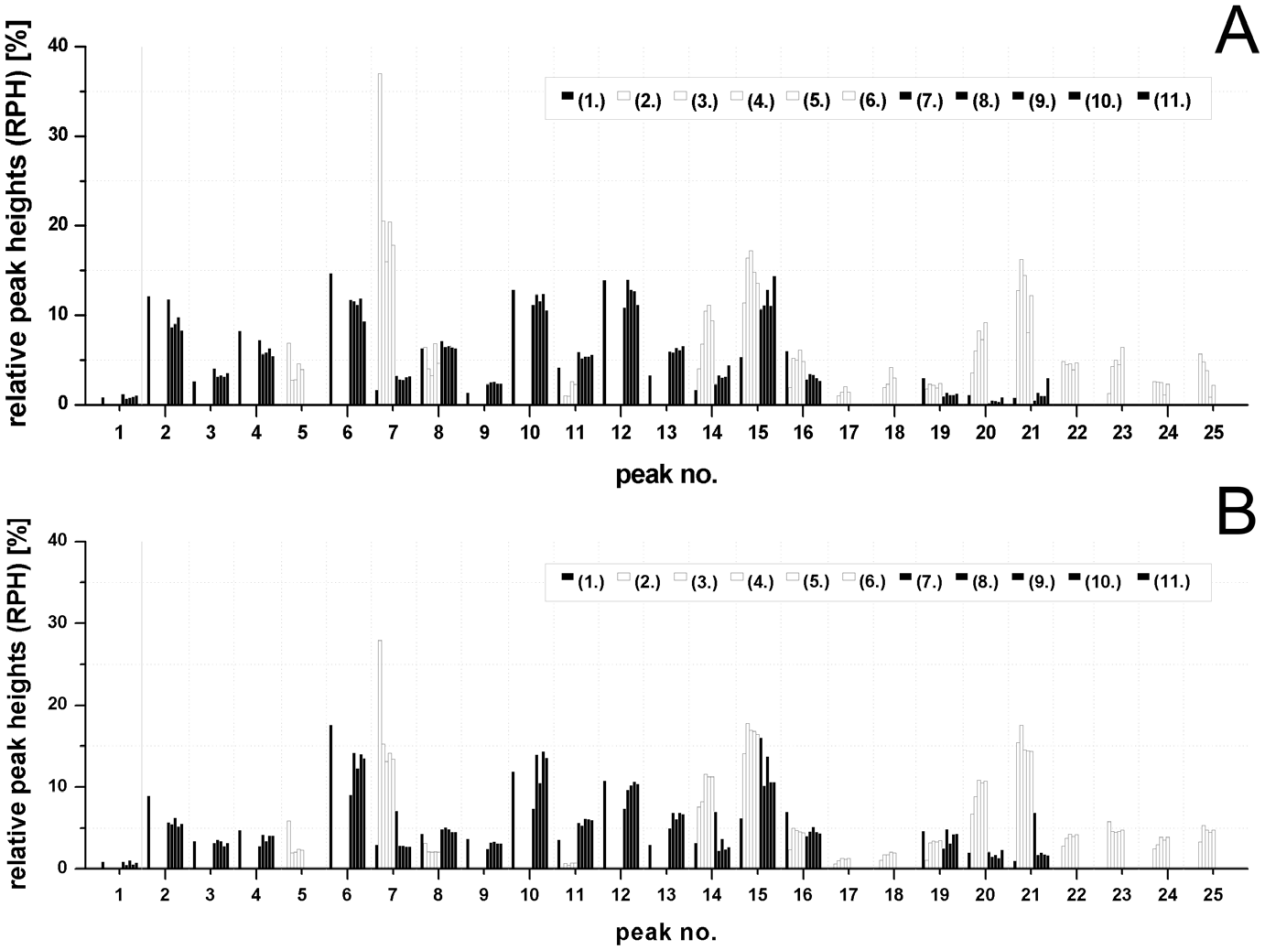
Relative peak heights of influenza A/PR/8/34 (*H1N1)* HA *N*-glycosylation patterns during virus adaptation. (A) for the isolate from RKI: In passages (1) and (7) to (11) virus was propagated in MDCK cell culture (▪). In passages (2) to (6) virus was propagated in Vero cell culture (□). Most of the 25 different major peaks are host cell-specific. Only the HA *N*-glycan structures represented by peak no. 7, 8, 11, 14 to 16 and 19 to 21 are present in virus samples from both host cells. (B) for the isolate from NIBSC: In passage (1) and (7) to (11) virus was propagated in MDCK cell culture (▪). In passage (2) to (6) virus was propagated in Vero cell culture (□). Most of the 25 different major peaks are host cell-specific. The HA *N*-glycan structures represented by peak no. 7, 8, 11, 14 to 16 and 19 to 21 are present in virus samples from both host cells.

All HA *N*-glycosylation patterns of MDCK cell-derived virus samples were similar. The same applied to the Vero cell-derived virus samples (figure 2C). In agreement with earlier studies the HA *N*-glycosylation pattern was strictly host cell-specific and changed significantly with the switch to the new host cells [28], [31]. However, of all Vero cell-derived HA *N*-glycosylation patterns for the RKI- as well as the NIBSC-strain passage 2 revealed the biggest differences in RPH (figure 3A). Here, the RPH of peak 5 and 7 was almost twice as high as for all subsequent passages in Vero cells. This is in agreement with the time series in Vero cells (figure S3) where these RPH almost doubled until 360 hpi. Furthermore, the low abundant glycan structure represented by peak 11 was missing in passage 2 of both strains (figure 2B, D; figure 3). This is most likely due to a drop below the detection limit. During the adaptation of the RKI-strain the height of peak 16 decreased by a factor of two in passage 2 (figure 3A, figure 2C), which was in agreement with the steady decrease of peak 16 during time course experiments (figure S3). Besides, peaks 17 and 18 were missing in passage 2 of the RKI-strain. In the time series in Vero cells these peaks represented low abundant structures with only 0.2 – 2.7% of TPH (figure S3). This probably indicates again a drop below the detection limit.

For each peak in both host-specific glycosylation patterns a comparison of the maximal SD in controls (biological replicates and the time courses) with the SD observed during adaptation experiments is shown in table S2. A more than 3-fold higher SD compared to the controls was considered significant. For the RKI-strain, during adaptation to Vero cells three peaks (11, 14 and 18) showed a more than 3-fold higher SD of RPH (table S2; Adaptation series *H1N1*, RKI) compared to the same peaks of the time series experiment (figure S3). Closer examination of the 15 main peaks of all MDCK cell-derived HA *N*-glycosylation patterns during back-adaptation revealed a more than 3-fold higher SD in RPH for peaks 6 and 14 (table S2; Adaptation series *H1N1*, RKI) compared to the respective time series and biological reproducibility experiments (figure S3). Of these peaks, only number 6, with an average RPH of 12.13%, represents a high abundant glycan structure above 5% of the TPH. For the NIBSC-strain, during forward adaptation, a 3-fold higher SD was found for the high abundant glycan structure represented by peak 14 (RPH >5%), as well as for another low abundant glycan structure represented by peak 19 (RPH <5%). Regarding the back-adaptation to MDCK cells the low abundant glycan structures of peaks 7 and 14, as well as the high abundant glycan structures of peak 6, 10 and 13 (RPH >5%) exhibited a more than 3-fold higher SD compared to controls.

Overall, however, a clear trend during the forward and backward adaptation process was not evident (figure 2C, figure 3). Furthermore, good reproducibility of the host cell-specific HA *N*-glycosylation pattern during virus adaptation to different host cell lines was demonstrated for both PR/8/34 influenza virus strains. Obviously, it is mainly the host cell line which determines the HA *N*-glycosylation patterns of a specific virus strain while the harvest time point or the viral adaptation status have only a minor impact on the respective RPH of glycan structures. Finally, the discrepancy of the HA *N*-glycosylation pattern for NIBSC- as well as the RKI-virus between the first passage in Vero cells (passage 2) and the patterns of all subsequent Vero cell adaptation passages (passages 3 – 6) can not completely be explained by differing harvest time points and remains to be further investigated.

### Changes in quasispecies composition

To investigate the impact of forward and backward adaptations in both cell lines segment 4 coding for the HA was sequenced for passage 1, 6 and 11. Focus was in particular on changes in potential HA *N*-glycosylation sites. The consensus sequences of segment 4 of the initial, MDCK cell-derived virus seeds from passage 1 are shown in the supplementary (figure S5). Regarding the consensus amino acid sequences of HA, the initial virus seeds from RKI and NIBSC differed in 7 amino acid positions. The differences comprise *K147-*, *A156E*, *E158K*, *I208L*, *R269M*, *F309Y* and *S398T* (RKI vs NIBSC, figure S5). For the RKI-strain, sequencing analysis clearly indicated that the virus population from the first initial MDCK passage 1 was uniform concerning RNA segment 4, i.e. only one virus variant was detected above the detection limit. In contrast segment 4 of the initial virus seed from NIBSC (passage 1) already comprised several virus variants. The following variants were detected with the specified frequencies (table 1, B): *Y24H* (22%), *T397A* (1.3%), *T397S* (0.6%), *D455Y* (21.4%) and *N460D* (12.2%).

**Table 1:** Changes of quasispecies composition on HA level of Influenza A/PR/8/34 (H1N1) (RKI, A) and (NIBSC, B) during virus adaptation.

A	DNA Level			Protein Level			Passage 1	Passage 6	Passage 11
	Base Substitution			Amino Acid Substitution			Population Ratio [%]	Population Ratio [%]	Population Ratio [%]
	C	1370	T	S	457	L	0	19	9
	A	1378	G	K	460	E	0	80	81
	no (initial seed virus)			no AA-substitutions			100	few reads	10

B	DNA Level			Protein Level			Passage 1	Passage 6	Passage 11
	Base Substitution			Amino Acid Substitution			Population Ratio [%]	Population Ratio [%]	Population Ratio [%]
	T	70	C	Y	24	H	22	0	0
	G	1183	A	V	395	M	0	41,5	11,3
	A	1189	G	T	397	A	1,3	0	0
	A	1189	T	T	397	S	0,6	0	5,4
	G	1363	T	D	455	Y	21,4	6,1	3,2
	G	1363	C	D	455	H	0	52	44,1
	A	1375	G	K	459	E	0	0	44,2
	A	1378	G	N	460	D	12,2	41,1	10,5

Passage 1 represents the MDCK cell adapted seed virus. Passage 6 represents the last of five successive virus passages in Vero cells. Passage 11 represents the last of five subsequent passages in MDCK cells, i.e. after final back-adaptation. 0% values correspond to below the detection limit. (A) In passage 6 and 11 of the PR/8/34 (RKI) the two substitutions at amino acid positions 457 and 460 are uncoupled. (B) In the first passage of the PR/8/34 (NIBSC) the two substitutions D455Y and N460D are uncoupled.

After complete adaptation to Vero cells (passage 6), 80% of the RKI-strain virus population carried amino acid substitution *K460E*, where a positively charged lysine was replaced by the negatively charged glutamic acid. Another population of 19% carried the *S457L* substitution, where polar serine was replaced by non-polar leucine (table 1A). These two substitutions were uncoupled. No single read was detected, which carried both substitutions. Hence, 99% of sequenced viruses carried either one of these two substitutions, indicating a crucial region of the HA for adaptation to efficient virus growth in Vero cells. Only single reads were detected that carried the original sequence of the first passage. This suggests that on the one hand, the initial virus did replicate in Vero cells, but only poorly. Considering the dilutions of the initial virus seed during the 5 Vero passages without any replication, its concentration would have been below 0.1 virion/mL in passage 6 and would most likely not have been detected at all. On the other hand, only a change in very few single amino acids in this HA-region is necessary to increase virus fitness for sufficient growth in Vero cells. In passage 6 of the adaptation of the NIBSC-strain a new variant carrying a *V395M* substitution was detected with a frequency of 41.5%, which introduced an additional sulfur containing residue into the HA2 peptide chain. Furthermore, the *D455Y* virus seed subpopulation dropped to 6.1%, but a new variant *D455H*, in which aspartic acid was substituted by the basic amino acid histidine, was detected that dominated the virus population with 52%. In addition, the *N460D* subpopulation increased to 41.1% (table 1B).

After back-adaptation of the RKI-strain to MDCK cells the dominating virus population (81%) still carried the *K460E* substitution in the HA. The minor subpopulation (19% after Vero-adaptation) carrying the *S457L* substitution decreased to 9% after back-adaptation. The remaining population of 10% represented the initial virus seed sequence from passage 1 (table 1A). This demonstrates that the *K460E* variant allows good virus replication in both cell lines. In contrast, the *S457L* variant seems to be less efficiently replicating in MDCK cells. These results, particularly the fitness of the *K460E* variant in MDCK as well as in Vero cells, strongly suggests that a mutation in this region was acquired, rather than an already existing virus subpopulation of the initial virus seed was selected. This mutation finally allowed sufficient virus replication in passage 2 of the adaptation series to reach an HA-titer of 1.4 HAU at 288 hpi. After back-adaptation of the NIBSC-strain to MDCK cells the *V395M*, the *D455Y*, the *D455H*, and the *N460D* variants decreased to 11.3%, 3.2%, 44.1%, and 10.5%, respectively. In contrast, the *T397S* variant that was not detected in passage 6, came-up again and made up for 5.4% in passage 11 (table 1B). This *T397S* substitution abolished the only difference of the consensus sequences in the HA2 chain from the two influenza viruses A/PR/8/34 (*H1N1*) strains from NIBSC and the RKI (figure S5). Furthermore a new variant carrying the *K459E* substitution was detected with a frequency of 44.2%.

All substitutions detected during adaptation of both A/PR/8/34 (*H1N1*) strains are located in the HA2 chain, neither inside nor in close proximity of any *N*-glycosylation site. They are, however, located in the inside of the HA trimer within or in close proximity to the fusion peptide pocket: within the subunits' contact site for the RKI-strain (figure S4A-D) and within the subunits' and monomer contact sites for the NIBSC-strain (figure S4E-H). This position belongs to the fusion subdomain [37]. Substitutions in this region of HA subunit contacts have been described to be crucial for the stability of the structure of the native protein. Among other factors, the optimal stability depends on pH or temperature [2], [38]-[40]. In this regard lower pH environments for instance require higher native structure stability, whereas elevated pH-values require less stable structure conformations to mediate membrane fusion. In 2010 Reed et al. demonstrated using recombinant H5N1 influenza viruses that substitutions within the fusion peptide pocket and the α-helix of HA2 alter the pH of activation of HA, which in term effects influenza virus pathogenicity as well as transmissibility in mallards [41]. Furthermore, Thoennes et al. showed that different substitutions at HA2 position 111 of a H3N2 influenza virus strain significantly effected fusion pH, suggesting, a key role of this residue for neutral pH structure stability [42]. Therefore, it remains to be investigated, whether the substitutions occurring during the adaptation processes from MDCK to Vero cell-based replication and back alter acid stability of HA [43], pH of activation, or membrane fusion [44]. Or, whether they simply counteract steric hindrance [45] caused by Vero-specific changes in HA *N*-glycosylation, e.g. on residues 28 and/or 40 in the stem region of HA to achieve low-pH conformation required for membrane fusion.

An alignment of the segment 4 sequence of the initial, MDCK cell-derived virus PR/8/34 (RKI) from passage 1 with the viral sequence published in 1981 [46] demonstrated homology with three exceptions. These included a substitution at position 146, where threonine was substituted by asparagine (*T146N*). In 2001, Schickli et al. [47] detected an asparagine instead of the threonine and lysine at this position (*T146N*, *K147-*). Furthermore, they described a second substitution *D204E* present in this virus strain (passage 1). The third substitution was located at position 158, where lysine was substituted by glutamic acid (*K158E*).

In summary, the genome sequences of RNA segment 4, coding for HA, are homogenous in the initial virus seed of the RKI-strain (passage 1). This suggests clearly that the virus seed consisted of only one population. The adaptation processes resulted in additional virus variants, due to mutations in the HA stem region. However, no potential HA *N*-glycosylation sites or amino acids in their close neighborhood were affected. In contrast, the initial virus seed population (passage 1) of the NIBSC-strain of the adaptation series comprises more subpopulations concerning the HA-encoding RNA segment 4 than the RKI virus seed. Previously, these two (theoretical identical) influenza virus seeds have been described to differ significantly in infection characteristics such as interferon (IFN) and apoptosis induction, expression of interferon stimulated genes, final virus yields [48], [49], and the activation of general host cell response [50], [51]: PR/8/34 from NIBSC was characterized to induce higher levels of IFN and Mx expression, to induce apoptosis earlier, and to reach lower final titers than the PR/8/34 from RKI. Seitz et al. hypothesized that two amino acid substitutions in the non-structural protein 1 (NS1) might be related to these differences. Our present findings of a set of slightly different virus variants in the NIBSC seed suggest that the lower average virus yields are due to a broadened quasispecies of the PR/8/34 (NIBSC) virus seed which comprises low-yield-virus variants. The heterogeneity of PR/8/34 (NIBSC) persisted during the whole adaptation processes: from passage 1 to 11, samples comprised multiple virus subpopulations. Thereby, sizes of subpopulations as well as selection and extinction of specific virus variants varied in dependence of adaptation pressure, i.e. the host. Similar findings were reported by Bull et al. for adaptation studies with bacteriophages. They observed the highest level of convergence for bacteriophages grown on the same host and furthermore finally deduced that most substitutions during forward and backward adaptation to different hosts were most probably driven by the adaptation pressure of host-switching [52].

A comparison of results of both adaptation experiments with influenza virus strains from NIBSC and from RKI shows one interesting similarity, the substitution of lysine by glutamic acid. In particular, the *K459E* variant from NIBSC that carries a deletion in the HA1 chain (*K147-*) corresponds to the *K460E* variant from the RKI. Moreover, also the substitutions *D455H* and *N460D* of the NIBSC strain are located in the same region of the HA2 subunit as the substitutions *S457L* and *K460E* of the RKI-strain.

For both influenza virus strains the amino acid substitutions in HA during virus adaptation are located exclusively in the HA2 chain (figure S4). Rott et al. [53] described the occurrence of virus variants after adaptation to MDCK cells, which exhibited elevated fusion pH, though these carried exclusively substitutions within the HA1 chain. In contrast, our experiments indicate a crucial role of the HA stem region for virus adaptation from MDCK to Vero cells. Here, inter-monomer or inter-subunit contact is mediated, suggesting HA-initiated fusion as a driving factor of adaptation pressure. Both adaptation series show a significant change of the HA *N*-glycosylation pattern with the change of host cell system. Additionally, both experiments demonstrate that only a small sequence adaptation is required for successful infection and fast growth to high titers in new host cell lines. These findings are in agreement with results of Wagner et al. and Klenk et al. [45], [54] who reported that *N*-glycans attached to the stem domain of HA efficiently regulate influenza A virus replication. The authors showed that a loss of *N*-glycans in the stem region results in increased pH-sensitivity of the virus and that these viruses are also temperature sensitive.

Taken together, our data show that a MDCK cell-adapted virus seed that had not been exposed to Vero cells before, hardly produces any virus progeny in Vero cells. Nevertheless, amino acid substitutions within the stem region of HA can rescue the virus population and ensure efficient virus replication in the new host. Interestingly, for both RKI- and NIBSC-derived virus strains the glycan pattern stabilized with the first passage in the new cell line, with all passage to passage SDs values below 8.5%. The faster release of virus particles and to begin with the further increase in HA-titers during passages two to six indicates that other factors are as well involved in the adaptation process, i.e. changes in the sequence of the viral genome resulting in improved fitness of virus subpopulations. In agreement with Schwarzer et al. the HA glycan profile of Vero cell-adapted virus shows a tendency towards smaller glycan structures compared to MDCK cell-derived viruses [31]. The fitness of these adapted virus variants during backward adaptation to MDCK cells varies – some variants as the *K460E* (RKI) replicate well and infect either cell line while others grow only poorly in one cell line (e.g. *S457L* (RKI) in MDCK cells). Whether the substitutions that occurred during the adaptation processes alter acid stability [43], pH of activation, or membrane fusion mechanism [44] of HA or simply counteract steric hindrance caused by Vero-specific changes in *N*-glycosylation on residues 28 and/or 40 in the stem region of HA for achieving a low-pH conformation, remains to be further elucidated. Finally it should be pointed out, that other factors besides the adaptation status to a host cell line contribute to HA *N*-glycosylation. Besides the host cell itself - as demonstrated by Schwarzer et al. [31] - one may have to include differences in cell culture media, cultivation systems and cultivation conditions, virus seed preparation, cell density and multiplicity of infection used in the virus production phase. Furthermore, little is known concerning the impact of changes in glycan profiles on properties of live and dead vaccines, i.e. safety and immunogenicity. Nevertheless, monitoring HA *N*-glycosylation patterns during vaccine production processes allows not only to investigate the impact of process modifications on antigen quality, but also offers a sensitive tool to evaluate consequences of unwanted process variations or process failures.

## Supporting Information

Figure S1 **Stability of HA** ***N*** **-glycosylation patterns of influenza A/PR/8/34 (** ***H1N1*** **, RKI).** (A) The shifted overlays of normalized capillary electropherograms demonstrate reproducible HA *N*-glycosylation patterns over 10 successive virus passages in MDCK cells. The relative fluorescence units (RFU) are plotted over the normalized migration time in basepairs (bp). All 10 patterns exhibit the same 15 numbered main peaks between 300 bp and 420 bp. (B) Relative peak heights (RPH) of the 15 main peaks. Standard deviations (error bars) for 10 successive virus passages, range between 0.25% and 1.14%. The corresponding relative standard deviations (RSD) of low abundant peaks, (each representing less than 5% of the total peak heights (TPH); numbered 1, 7, 9, 14, 16) range between 8.16% and 23.64%, while the RSD for all high abundant peaks (each representing more than 5% of TPH) range between 4.16% and 7.65%.
(TIF)

Figure S2 **Impact of harvest time point on the HA** ***N*** **-glycosylation pattern of MDCK cell-derived influenza A PR/8/34 (** ***H1N1*** **, RKI).** (A) Relative fluorescence units (RFU) are plotted over the normalized migration time in basepairs (bp). The shifted overlay of normalized capillary electropherograms demonstrates an overall stability of the HA *N*-glycosylation pattern within the range from 24 hpi to 96 hpi. Both harvest time points exhibited the same 15 numbered main peaks (peak no.: 1-4, 6-16, cf. figure 1) with migration times between 300 bp and 420 bp. (B) The relative peak heights (RPH) of the 15 dominating peaks (no.: 1-4, 6-16) are represented by grey (24 hpi) or white (96 hpi) columns; corresponding average values (⁃) with the respective standard deviations (error bars) are indicated in black.
(TIF)

Figure S3 **Impact of harvest time point on the HA** ***N*** **-glycosylation pattern of Vero cell-derived influenza A PR/8/34 (** ***H1N1*** **, RKI).** (A) Relative fluorescence units (RFU) are plotted over the normalized migration time in basepairs (bp). The shifted overlay of normalized capillary electropherograms demonstrates an overall stability of the HA *N*-glycosylation pattern from 48 hpi to 360 hpi. The harvest time point has an impact on the RPH of the 16 numbered main peaks (peak no.: 5, 7, 8, 11, 15-25), exhibiting normalized migration times between 220 bp and 380 bp. (B) For each harvest time point, the relative peak heights (RPH) of all 16 dominating peaks (no.: 5, 7, 8, 11, 15-25) are represented by a column. Corresponding average values (⁃) and standard deviations (error bars) are indicated in grey.
(TIF)

Figure S4 **Localization of substitutions during adaptation within the 3D HA-stucture.** Structures are disolayed in a cartoon diagram with potential HA *N*-glycosylation sites highlighted by red space filled residues. (A, B, C, D) Influenza A/PR/8/34 (*H1N1*, RKI), (E, F, G, H) influenza A/PR/8/34 (*H1N1*, NIBSC). Trimeric (A-C, E-G) and monomeric (D, H) HA molecules. (A-D) The HA1 chains are colored in pink, green and brown; the HA2 chains are colored in blue, grey and orange. The *K460E* mutation is highlighted in yellow, the *S457L* substitution by white space-filled residues. (A) Bottom (B) side and (C) top view. (D) indicates the close proximity within the monomer of these two substitutions, which are one helix turn apart from each other. (E-H) For the isolate from NIBSC the HA1 chains are colored in pink, purple and green; the HA2 chains are colored in turquoise, yellow and orange. The substitutions already present in the virus seed are highlighted by grey (*Y24H*), yellow (*D455Y*) and pink (*N460D*) space-filled residues. Substitutions occurring during virus adaptation are indicated by white (*V395M*, *T397S*) or yellow (*D455H*) space-filled residues. (E) Top, (F, H) side, (G) bottom view. The PDB entry 1RU7 and PyMOL (v0.99, DeLano Scientific LLC, California, USA) software was used for structure display.
(TIF)

Figure S5 **Alignment of HA amino acid consensus sequences of two closely related influenza viruses A/PR/8/34 (** ***H1N1*** **).** The virus seed of RKI (Amp. 3138) corresponds to a homogeneous population. Substitutions in the sequence during the virus adaptation processes are indicated in red. In contrast, the virus seed from NIBSC (#06/114) comprises various virus variants; substitutions in the sequence are indicated in green. The positions of substitutions, acquired during the adaptation processes are indicated in blue. The amino acid assembly was performed at http://services.uniprot.org/clustalw.
(TIF)

Table S1 **Accession numbers for influenza A virus PR/8/34 sequences during adaptation from MDCK to Vero cells and back.** The original virus seed was either purchased from the National Institute for Biological Standards and Control (NIBSC) or the Robert Koch Institute (RKI). The first virus passages, produced in MDCK cell culture (M1), served as virus seed for the first pasages in Vero cells. The last of five consecutive Vero cell-derived virus passages (V5) served as seed for five consecutive MDCK cell-derived virus passages, of which M6 represents the last. All sequences were generated by pyrosequencing and deposited in the GISAID EpiFlu database (www.gisaid.org).
(PDF)

Table S2 **Overview of relative peak heights (RPH) averages and according standard (SD) and relative standard deviations (RSD).** The average RPH and the respective SD and RSD of each peak (no. 1 - 25) within each experiment (control 1: pattern stability for ten consecutive virus passages; control 2: reproducibility in Vero or MDCK time series; adaptation series of *H1N1* from RKI and adaptation series of *H1N1* from NIBSC) are listed. The factors indicate x–fold increase (>1) or dercease (<1) of respective deviations observed during adaptation compared to the maximal deviation during controls. Factors of > 3 are defined as significantly influenced during adaptation and highlighted in blue bold numbers.
(PDF)

## References

[1] Webster RG, Bean WJ, Gorman OT, Chambers TM, Kawaoka Y (1992). Evolution and ecology of influenza A viruses.. Microbiol Rev.

[2] Lin YP, Wharton SA, Martin J, Skehel JJ, Wiley DC (1997). Adaptation of egg-grown and transfectant influenza viruses for growth in mammalian cells: selection of hemagglutinin mutants with elevated pH of membrane fusion.. Virology.

[3] Hirst GK (1947). Studies on the Mechanism of Adaptation of Influenza Virus to Mice.. J Exp Med.

[4] Webster RG, Laver WG, Air GM, Schild GC (1982). Molecular mechanisms of variation in influenza viruses.. Nature.

[5] Hensley SE, Das SR, Bailey AL, Schmidt LM, Hickman HD (2009). Hemagglutinin Receptor Binding Avidity Drives Influenza A Virus Antigenic Drift.. Science.

[6] Rogers GN, Paulson JC (1983). Receptor determinants of human and animal influenza virus isolates: differences in receptor specificity of the H3 hemagglutinin based on species of origin.. Virology.

[7] de Wit E, Munster VJ, van Riel D, Beyer WEP, Rimmelzwaan GF (2010). Molecular Determinants of Adaptation of Highly Pathogenic Avian Influenza H7N7 Viruses to Efficient Replication in the Human Host.. Journal of Virology.

[8] Skehel JJ, Wiley DC (2000). Receptor binding and membrane fusion in virus entry: the influenza hemagglutinin.. Annu Rev Biochem.

[9] Wrigley NG (1979). Electron microscopy of influenza virus.. Br Med Bull.

[10] Johansson BE, Bucher DJ, Kilbourne ED (1989). Purified influenza virus hemagglutinin and neuraminidase are equivalent in stimulation of antibody response but induce contrasting types of immunity to infection.. J Virol.

[11] Rudd PM, Dwek RA (1997). Glycosylation: Heterogeneity and the 3D Structure of Proteins.. Critical Reviews in Biochemistry and Molecular Biology.

[12] Abe Y, Takashita E, Sugawara K, Matsuzaki Y, Muraki Y (2004). Effect of the Addition of Oligosaccharides on the Biological Activities and Antigenicity of Influenza A/H3N2 Virus Hemagglutinin.. Journal of Virology.

[13] Saito T, Nakaya Y, Suzuki T, Ito R, Saito T (2004). Antigenic alteration of influenza B virus associated with loss of a glycosylation site due to host-cell adaptation.. Journal of Medical Virology.

[14] Chen Z, Aspelund A, Jin H (2008). Stabilizing the glycosylation pattern of influenza B hemagglutinin following adaptation to growth in eggs.. Vaccine.

[15] Opitz L, Zimmermann A, Lehmann S, Genzel Y, Lubben H (2008). Capture of cell culture-derived influenza virus by lectins: Strain independent, but host cell dependent.. Journal of Virological Methods.

[16] de Vries RP, de Vries E, Bosch BJ, de Groot RJ, Rottier PJM (2010). The influenza A virus hemagglutinin glycosylation state affects receptor-binding specificity.. Virology.

[17] Domingo E, Baranowski E, Ruiz-Jarabo CM, Martin-Hernandez AM, Saiz JC (1998). Quasispecies structure and persistence of RNA viruses.. Emerg Infect Dis.

[18] Domingo E, Sabo D, Taniguchi T, Weissmann C (1978). Nucleotide sequence heterogeneity of an RNA phage population.. Cell.

[19] Biebricher CK, Eigen M (2006). What is a quasispecies?. Curr Top Microbiol Immunol.

[20] Scholtissek C (1995). Molecular evolution of influenza viruses.. Virus Genes.

[21] Kilbourne ED (1969). Future influenza vaccines and the use of genetic recombinants.. Bull World Health Organ.

[22] Steinhauer DA, Holland JJ (1987). Rapid evolution of RNA viruses.. Annu Rev Microbiol.

[23] Domingo E, Martin V, Perales C, Grande-Perez A, Garcia-Arriaza J (2006). Viruses as quasispecies: biological implications.. Curr Top Microbiol Immunol.

[24] Lauring AS, Andino R (2010). Quasispecies theory and the behavior of RNA viruses.. PLoS Pathog.

[25] Gerdil C (2003). The annual production cycle for influenza vaccine.. Vaccine.

[26] Robertson JS, Nicolson C, Harvey R, Johnson R, Major D (2011). The development of vaccine viruses against pandemic A(H1N1) influenza.. Vaccine.

[27] Genzel Y (2004). Metabolism of MDCK cells during cell growth and influenza virus production in large-scale microcarrier culture.. Vaccine.

[28] Genzel Y, Dietzsch C, Rapp E, Schwarzer J, Reichl U (2010). MDCK and Vero cells for influenza virus vaccine production: a one-to-one comparison up to lab-scale bioreactor cultivation.. Applied Microbiology and Biotechnology.

[29] Kalbfuss B, Knochlein A, Krober T, Reichl U (2008). Monitoring influenza virus content in vaccine production: Precise assays for the quantitation of hemagglutination and neuraminidase activity.. Biologicals.

[30] Schwarzer J, Rapp E, Reichl U (2008). N-glycan analysis by CGE-LIF: Profiling influenza A virus hemagglutininN-glycosylation during vaccine production.. Electrophoresis.

[31] Schwarzer J, Rapp E, Hennig R, Genzel Y, Jordan I (2009). Glycan analysis in cell culture-based influenza vaccine production: Influence of host cell line and virus strain on the glycosylation pattern of viral hemagglutinin.. Vaccine.

[32] Ruhaak LR, Hennig R, Huhn C, Borowiak M, Dolhain RJ (2010). Optimized workflow for preparation of APTS-labeled N-glycans allowing high-throughput analysis of human plasma glycomes using 48-channel multiplexed CGE-LIF.. J Proteome Res.

[33] Hoper D, Hoffmann B, Beer M (2009). Simple, sensitive, and swift sequencing of complete H5N1 avian influenza virus genomes.. J Clin Microbiol.

[34] Leifer I, Hoffmann B, Hoper D, Bruun Rasmussen T, Blome S (2010). Molecular epidemiology of current classical swine fever virus isolates of wild boar in Germany.. J Gen Virol.

[35] Hoper D, Hoffmann B, Beer M (2011). A comprehensive deep sequencing strategy for full-length genomes of influenza a.. PLoS ONE.

[36] Wiley G, Macmil S, Qu C, Wang P, Xing Y (2009). Methods for generating shotgun and mixed shotgun/paired-end libraries for the 454 DNA sequencer.. Curr Protoc Hum Genet Chapter 18: Unit18.

[37] Gamblin SJ (2004). The Structure and Receptor Binding Properties of the 1918 Influenza Hemagglutinin.. Science.

[38] Ruigrok RW, Wrigley NG, Calder LJ, Cusack S, Wharton SA (1986). Electron microscopy of the low pH structure of influenza virus haemagglutinin.. EMBO J.

[39] Ruigrok RW, Martin SR, Wharton SA, Skehel JJ, Bayley PM (1986). Conformational changes in the hemagglutinin of influenza virus which accompany heat-induced fusion of virus with liposomes.. Virology.

[40] Doms RW, Gething MJ, Henneberry J, White J, Helenius A (1986). Variant influenza virus hemagglutinin that induces fusion at elevated pH.. J Virol.

[41] Reed ML, Bridges OA, Seiler P, Kim JK, Yen HL (2010). The pH of activation of the hemagglutinin protein regulates H5N1 influenza virus pathogenicity and transmissibility in ducks.. J Virol.

[42] Thoennes S, Li ZN, Lee BJ, Langley WA, Skehel JJ (2008). Analysis of residues near the fusion peptide in the influenza hemagglutinin structure for roles in triggering membrane fusion.. Virology.

[43] Brown JD, Swayne DE, Cooper RJ, Burns RE, Stallknecht DE (2007). Persistence of H5 and H7 avian influenza viruses in water.. Avian Dis.

[44] Reed ML, Yen HL, DuBois RM, Bridges OA, Salomon R (2009). Amino Acid Residues in the Fusion Peptide Pocket Regulate the pH of Activation of the H5N1 Influenza Virus Hemagglutinin Protein.. Journal of Virology.

[45] Wagner R, Heuer D, Wolff T, Herwig A, Klenk HD (2002). N-Glycans attached to the stem domain of haemagglutinin efficiently regulate influenza A virus replication.. J Gen Virol.

[46] Winter G, Fields S, Brownlee GG (1981). Nucleotide sequence of the haemagglutinin gene of a human influenza virus H1 subtype.. Nature.

[47] Schickli JH, Flandorfer A, Nakaya T, Martinez-Sobrido L, Garcia-Sastre A (2001). Plasmid-only rescue of influenza A virus vaccine candidates.. Philos Trans R Soc Lond B Biol Sci.

[48] Seitz C, Frensing T, Hoper D, Kochs G, Reichl U (2010). High yields of influenza A virus in Madin-Darby canine kidney cells are promoted by an insufficient interferon-induced antiviral state.. J Gen Virol.

[49] Schulze-Horsel J, Schulze M, Agalaridis G, Genzel Y, Reichl U (2009). Infection dynamics and virus-induced apoptosis in cell culture-based influenza vaccine production-Flow cytometry and mathematical modeling.. Vaccine.

[50] Heynisch B, Frensing T, Heinze K, Seitz C, Genzel Y (2010). Differential activation of host cell signalling pathways through infection with two variants of influenza A/Puerto Rico/8/34 (H1N1) in MDCK cells.. Vaccine.

[51] Vester D, Rapp E, Kluge S, Genzel Y, Reichl U (2010). Virus-host cell interactions in vaccine production cell lines infected with different human influenza A virus variants: a proteomic approach.. J Proteomics.

[52] Bull JJ, Badgett MR, Wichman HA, Huelsenbeck JP, Hillis DM (1997). Exceptional convergent evolution in a virus.. Genetics.

[53] Rott R, Orlich M, Klenk HD, Wang ML, Skehel JJ (1984). Studies on the adaptation of influenza viruses to MDCK cells.. EMBO J.

[54] Klenk HD, Wagner R, Heuer D, Wolff T (2002). Importance of hemagglutinin glycosylation for the biological functions of influenza virus.. Virus Res.

