# Supplementary figures and images for: Impact of Host Cell Line Adaptation on Quasispecies Composition and Glycosylation of Influenza A Virus Hemagglutinin
Source: PLoS One. 2011 Dec 7;6(12):e27989. doi: 10.1371/journal.pone.0027989 (PMC3233551; doi:10.1371/journal.pone.0027989)

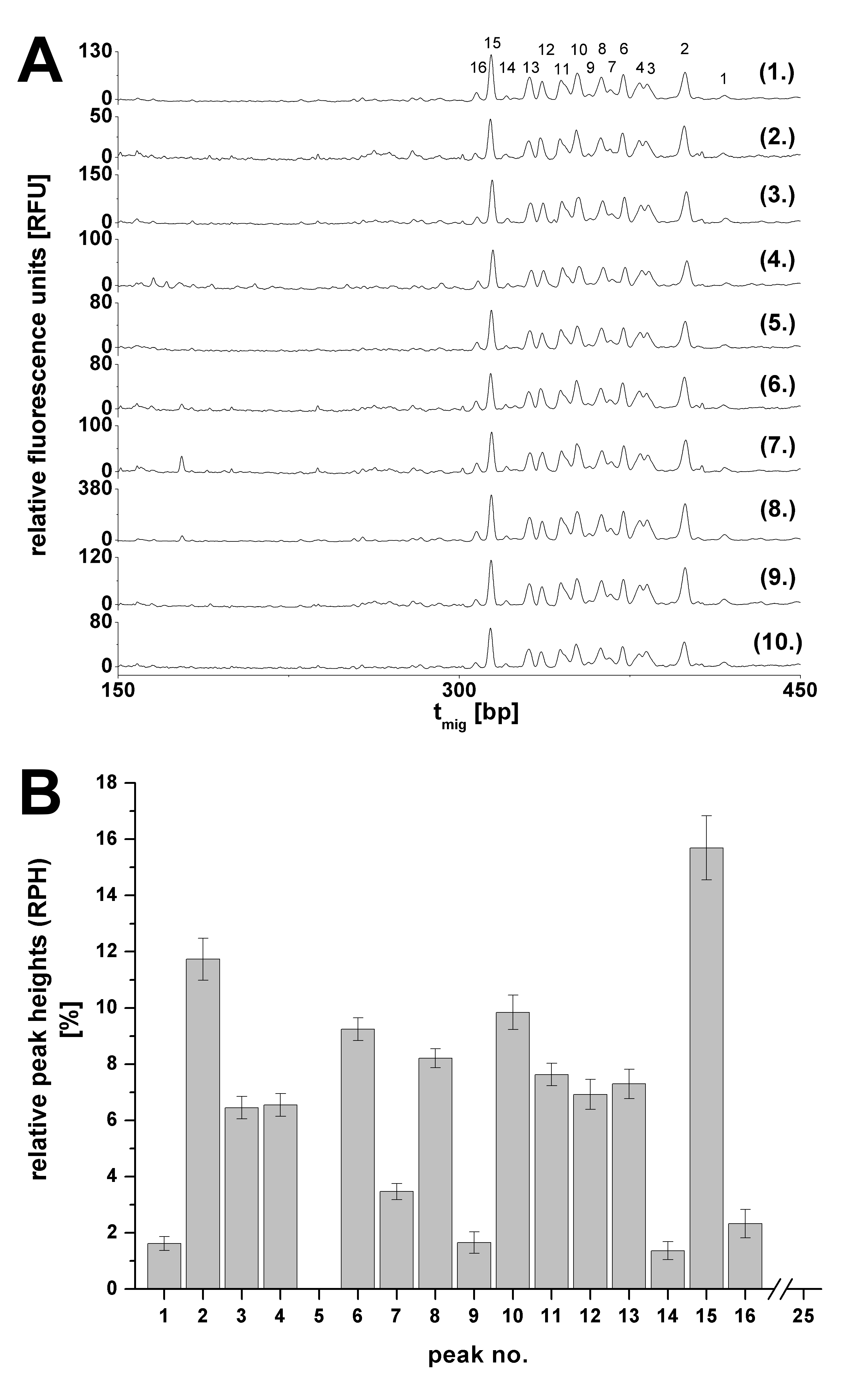

Supplement: Figure S1 — Stability of HA N -glycosylation patterns of influenza A/PR/8/34 ( H1N1 , RKI). (A) The shifted overlays of normalized capillary electropherograms demonstrate reproducible HA N-glycosylation patterns over 10 successive virus passages in MDCK cells. The relative fluorescence units (RFU) are plotted over the normalized migration time in basepairs (bp). All 10 patterns exhibit the same 15 numbered main peaks between 300 bp and 420 bp. (B) Relative peak heights (RPH) of the 15 main peaks. Standard deviations (error bars) for 10 successive virus passages, range between 0.25% and 1.14%. The corresponding relative standard deviations (RSD) of low abundant peaks, (each representing less than 5% of the total peak heights (TPH); numbered 1, 7, 9, 14, 16) range between 8.16% and 23.64%, while the RSD for all high abundant peaks (each representing more than 5% of TPH) range between 4.16% and 7.65%. (TIF) [file pone.0027989.s001.tif]

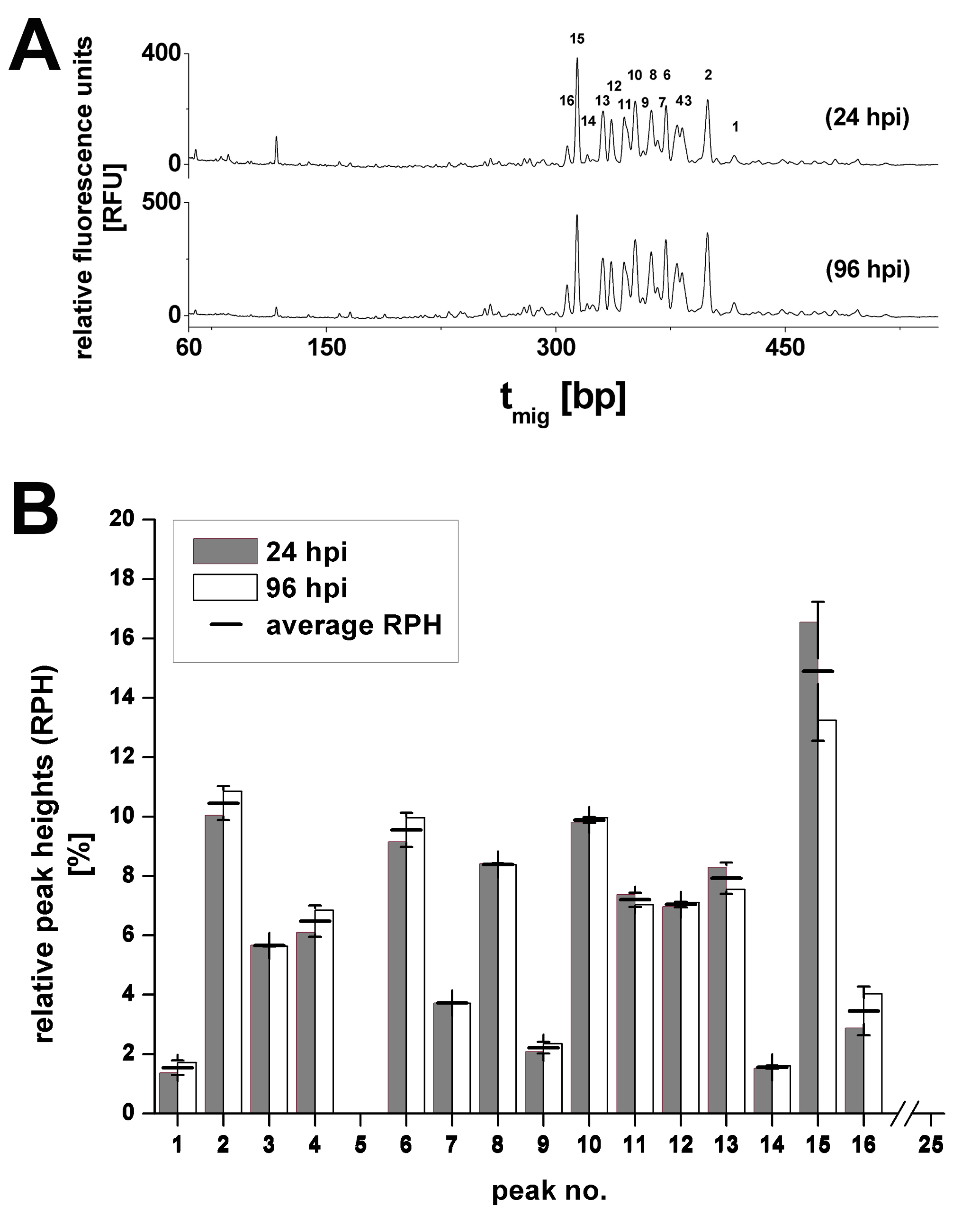

Supplement: Figure S2 — Impact of harvest time point on the HA N -glycosylation pattern of MDCK cell-derived influenza A PR/8/34 ( H1N1 , RKI). (A) Relative fluorescence units (RFU) are plotted over the normalized migration time in basepairs (bp). The shifted overlay of normalized capillary electropherograms demonstrates an overall stability of the HA N-glycosylation pattern within the range from 24 hpi to 96 hpi. Both harvest time points exhibited the same 15 numbered main peaks (peak no.: 1-4, 6-16, cf. figure 1) with migration times between 300 bp and 420 bp. (B) The relative peak heights (RPH) of the 15 dominating peaks (no.: 1-4, 6-16) are represented by grey (24 hpi) or white (96 hpi) columns; corresponding average values (⁃) with the respective standard deviations (error bars) are indicated in black. (TIF) [file pone.0027989.s002.tif]

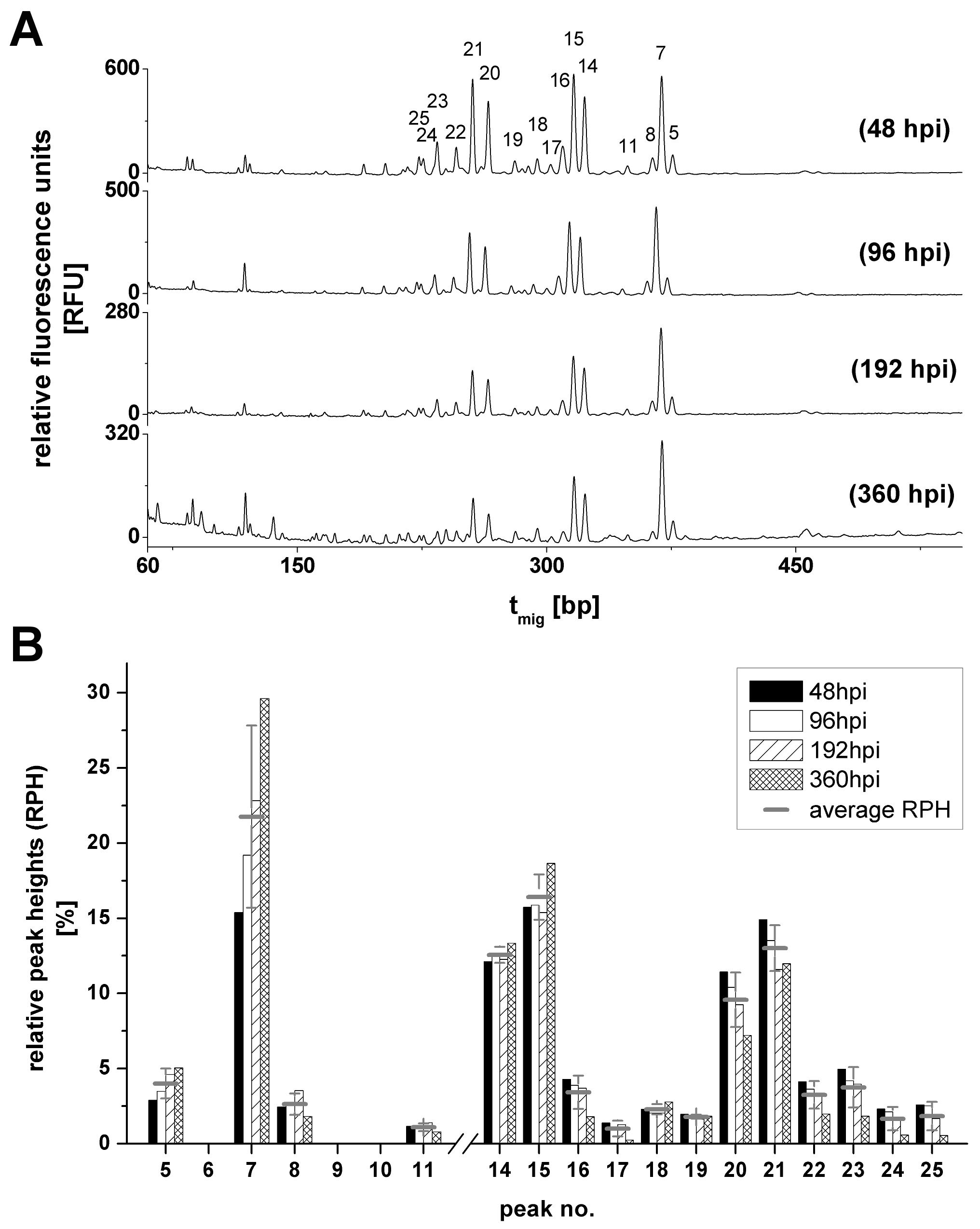

Supplement: Figure S3 — Impact of harvest time point on the HA N -glycosylation pattern of Vero cell-derived influenza A PR/8/34 ( H1N1 , RKI). (A) Relative fluorescence units (RFU) are plotted over the normalized migration time in basepairs (bp). The shifted overlay of normalized capillary electropherograms demonstrates an overall stability of the HA N-glycosylation pattern from 48 hpi to 360 hpi. The harvest time point has an impact on the RPH of the 16 numbered main peaks (peak no.: 5, 7, 8, 11, 15-25), exhibiting normalized migration times between 220 bp and 380 bp. (B) For each harvest time point, the relative peak heights (RPH) of all 16 dominating peaks (no.: 5, 7, 8, 11, 15-25) are represented by a column. Corresponding average values (⁃) and standard deviations (error bars) are indicated in grey. (TIF) [file pone.0027989.s003.tif]

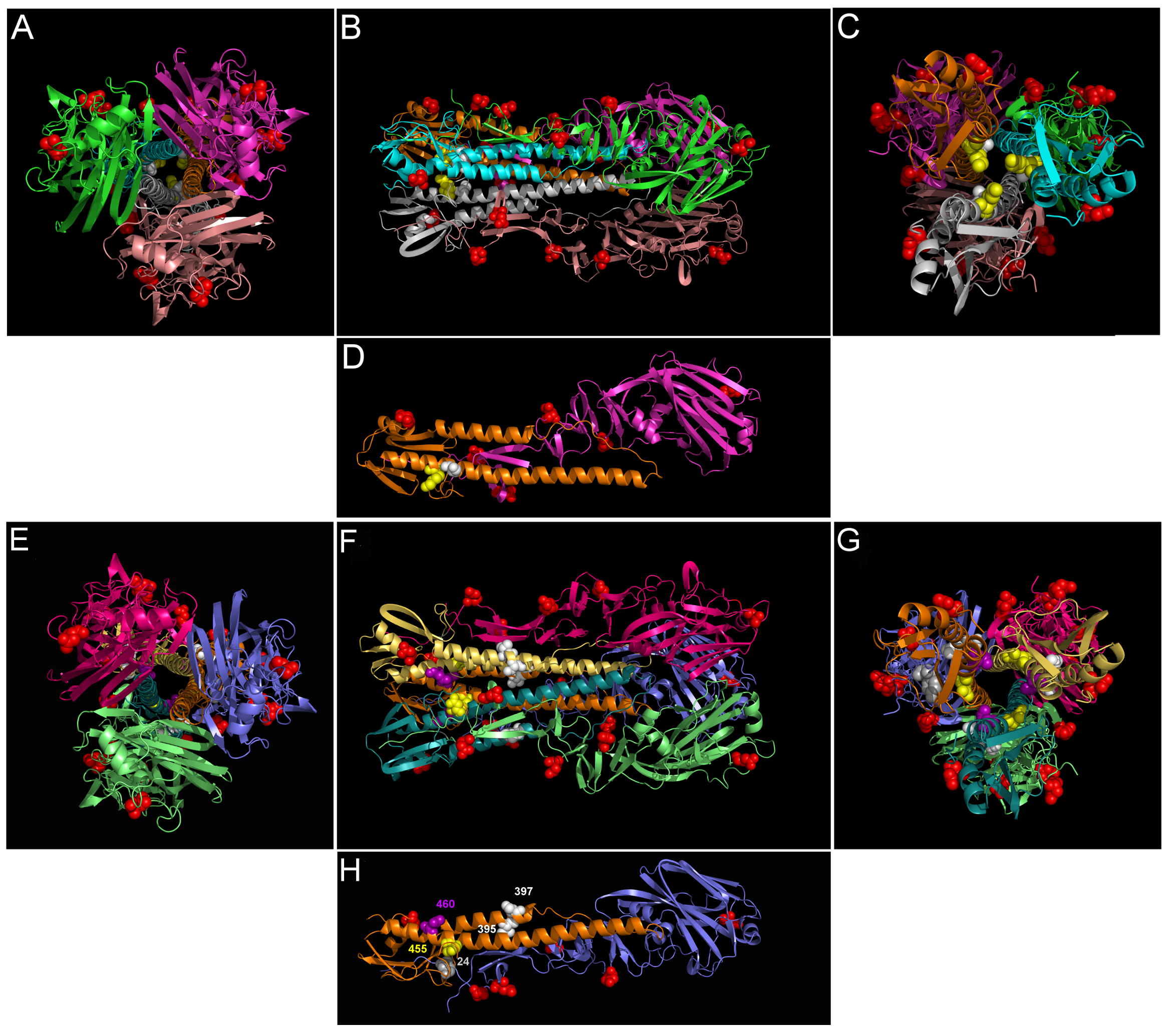

Supplement: Figure S4 — Localization of substitutions during adaptation within the 3D HA-stucture. Structures are disolayed in a cartoon diagram with potential HA N-glycosylation sites highlighted by red space filled residues. (A, B, C, D) Influenza A/PR/8/34 (H1N1, RKI), (E, F, G, H) influenza A/PR/8/34 (H1N1, NIBSC). Trimeric (A-C, E-G) and monomeric (D, H) HA molecules. (A-D) The HA1 chains are colored in pink, green and brown; the HA2 chains are colored in blue, grey and orange. The K460E mutation is highlighted in yellow, the S457L substitution by white space-filled residues. (A) Bottom (B) side and (C) top view. (D) indicates the close proximity within the monomer of these two substitutions, which are one helix turn apart from each other. (E-H) For the isolate from NIBSC the HA1 chains are colored in pink, purple and green; the HA2 chains are colored in turquoise, yellow and orange. The substitutions already present in the virus seed are highlighted by grey (Y24H), yellow (D455Y) and pink (N460D) space-filled residues. Substitutions occurring during virus adaptation are indicated by white (V395M, T397S) or yellow (D455H) space-filled residues. (E) Top, (F, H) side, (G) bottom view. The PDB entry 1RU7 and PyMOL (v0.99, DeLano Scientific LLC, California, USA) software was used for structure display. (TIF) [file pone.0027989.s004.tif]

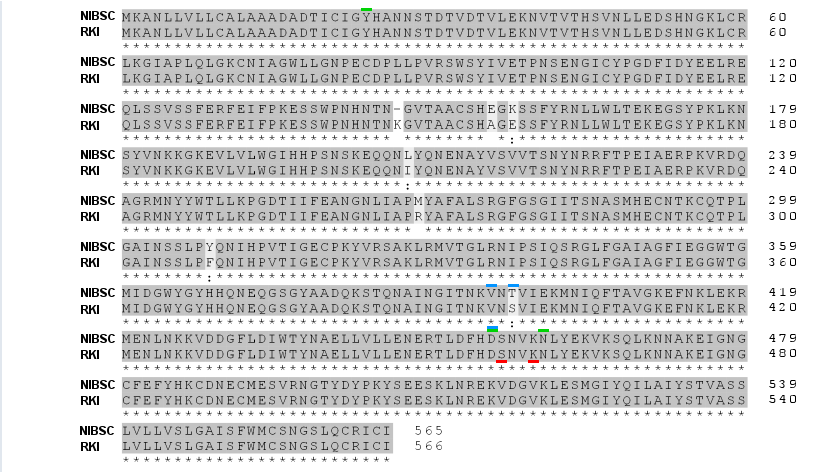

Supplement: Figure S5 — Alignment of HA amino acid consensus sequences of two closely related influenza viruses A/PR/8/34 ( H1N1 ). The virus seed of RKI (Amp. 3138) corresponds to a homogeneous population. Substitutions in the sequence during the virus adaptation processes are indicated in red. In contrast, the virus seed from NIBSC (#06/114) comprises various virus variants; substitutions in the sequence are indicated in green. The positions of substitutions, acquired during the adaptation processes are indicated in blue. The amino acid assembly was performed at http://services.uniprot.org/clustalw. (TIF) [file pone.0027989.s005.tif]
