# Supplementary material for: Impact of Host Cell Line Adaptation on Quasispecies Composition and Glycosylation of Influenza A Virus Hemagglutinin
Source: PLoS One. 2011 Dec 7;6(12):e27989. doi: 10.1371/journal.pone.0027989 (PMC3233551; doi:10.1371/journal.pone.0027989)
Supplement: Table S1 — Accession numbers for influenza A virus PR/8/34 sequences during adaptation from MDCK to Vero cells and back. The original virus seed was either purchased from the National Institute for Biological Standards and Control (NIBSC) or the Robert Koch Institute (RKI). The first virus passages, produced in MDCK cell culture (M1), served as virus seed for the first pasages in Vero cells. The last of five consecutive Vero cell-derived virus passages (V5) served as seed for five consecutive MDCK cell-derived virus passages, of which M6 represents the last. All sequences were generated by pyrosequencing and deposited in the GISAID EpiFlu database (www.gisaid.org). (PDF) [file pone.0027989.s006.pdf]

table S1: Accession numbers for influenza A virus PR/8/34 sequences during adaptation from MDCK to Vero cells and back. The original virus seed was either purchased from the National Institute for Biological Standards and Control (NIBSC) or the Robert Koch Institute (RKI). The first virus passages, produced in MDCK cell culture (M1), served as virus seed for the first pasages in Vero cells. The last of five consecutive Vero cell-derived virus passages (V5) served as seed for five consecutive MDCK cell-derived virus passages, of which M6 represents the last. All sequences were generated by pyrosequencing and deposited in the GISAID EpiFlu database (www.gisaid.org).

Segment_Ids	Isolate_Name	Passage_History	Seq_Id (HA)
EPI304412	A/MDCK/Germany/[A/Puerto Rico/8/1934]-RKI-M1 (H1N1)	P5	utf_rki-m1-ges_unambig_HA_Segment4_00001 length=1769 numreads=9121
EPI304420	A/MDCK/Germany/[A/Puerto Rico/8/1934]-RKI-V5 (H1N1)	P10	utf_rki-v5-ges_unambig_HA_Segment4_00001 length=1763 numreads=3748
EPI304428	A/MDCK/Germany/[A/Puerto Rico/8/1934]-RKI-M6 (H1N1)	P15	mutf_rki-m6-ges_unambig_HA_Segment4_00001, 1..1761 length=1760 numreads=2824
EPI304436	A/MDCK/Germany/[A/Puerto Rico/8/1934]-NIBSC-M1 (H1N1)	P4	mutf_nibsc-m1-ges_unambig_HA_Segment4_00001 length=1762 numreads=4279
EPI304444	A/MDCK/Germany/[A/Puerto Rico/8/1934]-NIBSC-V5 (H1N1)	P9	ctf_nibsc-v5-ges_unambig_HA_Segment4_00001 length=1760 numreads=3493
EPI304452	A/MDCK/Germany/[A/Puerto Rico/8/1934]-NIBSC-M6 (H1N1)	P14	mutf_nibsc-m6-ges_unambig_HA_Segment4_00001, 1..1759 length=1760 numreads=8589
