# Supplementary material for: Impact of Host Cell Line Adaptation on Quasispecies Composition and Glycosylation of Influenza A Virus Hemagglutinin
Source: PLoS One. 2011 Dec 7;6(12):e27989. doi: 10.1371/journal.pone.0027989 (PMC3233551; doi:10.1371/journal.pone.0027989)
Supplement: Table S2 — Overview of relative peak heights (RPH) averages and according standard (SD) and relative standard deviations (RSD). The average RPH and the respective SD and RSD of each peak (no. 1 - 25) within each experiment (control 1: pattern stability for ten consecutive virus passages; control 2: reproducibility in Vero or MDCK time series; adaptation series of H1N1 from RKI and adaptation series of H1N1 from NIBSC) are listed. The factors indicate x–fold increase (>1) or dercease (<1) of respective deviations observed during adaptation compared to the maximal deviation during controls. Factors of > 3 are defined as significantly influenced during adaptation and highlighted in blue bold numbers. (PDF) [file pone.0027989.s007.pdf]

**Table S2: Overview of relative peak heights (RPH) averages and according standard (SD) and relative standard deviations (RSD).** The average RPH and the respective SD and RSD of each peak (no. 1 - 25) within each experiment (control 1: pattern stability for ten consecutive virus passages; control 2: reproducibility in Vero or MDCK time series; adaptation series of *H1N1* from RKI and adaptation series of *H1N1* from NIBSC) are listed. The factors indicate x–fold increase (> 1) or dercease (< 1) of respective deviations observed during adaptation compared to the maximal deviation during controls. Factors of > 3 are defined as significantly influenced during adaptation and highlighted in blue bold numbers.

| Host Cell Line | Peak No. | Pattern Stability (Control 1)* | | | Reproducibility in Time Series (Control 2)° | | | max. deviation of controls | | Adaptation Series *H1N1 (Amp. 3138,* RKI)[#] | | | | | Adaptation Series *H1N1* (#06/114, NIBSC)[#] | | | | |
|---|---|---|---|---|---|---|---|---|---|---|---|---|---|---|---|---|---|---|---|
| | | $RPH_{average}$ [%] | SD [%] | RSD [%] | $RPH_{average}$ [%] | SD [%] | RSD [%] | SD [%] | RSD [%] | $RPH_{average}$ [%] | SD [%] | RSD [%] | $factor_{RSD}$ | $factor_{SD}$ | $RPH_{average}$ [%] | SD [%] | RSD [%] | $factor_{RSD}$ | $factor_{SD}$ |
| **Vero** | **5** | n.a. | n.a. | n.a. | 4.01 | 0.99 | 24.83 | 0.99 | 24.83 | 4.18 | 1.72 | 41.21 | 1.66 | 1.73 | 2.92 | 1.65 | 56.73 | 2.29 | 1.66 |
| | **7** | n.a. | n.a. | n.a. | 21.76 | 6.06 | 27.83 | 6.06 | 27.83 | 22.34 | 8.40 | 37.62 | 1.35 | 1.39 | 16.77 | 6.29 | 37.52 | 1.35 | 1.04 |
| | **8** | n.a. | n.a. | n.a. | 2.63 | 0.71 | 27.08 | 0.71 | 27.08 | 5.01 | 1.54 | 30.79 | 1.14 | 2.17 | 2.28 | 0.48 | 20.93 | 0.77 | 0.67 |
| | **11** | n.a. | n.a. | n.a. | 1.09 | 0.26 | 23.97 | 0.26 | 23.97 | 1.35 | 1.06 | 78.51 | **3.28** | **4.05** | 0.50 | 0.30 | 59.93 | 2.50 | 1.16 |
| | **14** | n.a. | n.a. | n.a. | 12.56 | 0.54 | 4.30 | 0.54 | 4.30 | 8.35 | 2.94 | 35.23 | **8.19** | **5.44** | 9.99 | 1.91 | 19.13 | **4.45** | **3.54** |
| | **15** | n.a. | n.a. | n.a. | 16.41 | 1.51 | 9.19 | 1.51 | 9.19 | 14.66 | 2.33 | 15.89 | 1.73 | 1.55 | 16.42 | 1.40 | 8.53 | 0.93 | 0.93 |
| | **16** | n.a. | n.a. | n.a. | 3.41 | 1.11 | 32.41 | 1.11 | 32.41 | 4.60 | 1.56 | 33.98 | 1.05 | 1.41 | 4.18 | 1.06 | 25.27 | 0.78 | 0.96 |
| | **17** | n.a. | n.a. | n.a. | 1.00 | 0.52 | 51.99 | 0.52 | 51.99 | 1.17 | 0.75 | 63.93 | 1.23 | 1.43 | 1.08 | 0.28 | 26.14 | 0.50 | 0.54 |
| | **18** | n.a. | n.a. | n.a. | 2.29 | 0.34 | 15.04 | 0.34 | 15.04 | 2.26 | 1.52 | 66.98 | **4.45** | **4.41** | 1.71 | 0.40 | 23.47 | 1.56 | 1.17 |
| | **19** | n.a. | n.a. | n.a. | 1.79 | 0.14 | 7.76 | 0.14 | 7.76 | 2.09 | 0.27 | 12.73 | 1.64 | 1.92 | 2.88 | 1.03 | 35.90 | **4.63** | **7.45** |
| | **20** | n.a. | n.a. | n.a. | 9.57 | 1.81 | 18.93 | 1.81 | 18.93 | 6.85 | 2.18 | 31.81 | 1.68 | 1.20 | 9.53 | 1.78 | 18.63 | 0.98 | 0.98 |
| | **21** | n.a. | n.a. | n.a. | 13.00 | 1.53 | 11.75 | 1.53 | 11.75 | 12.72 | 3.06 | 24.08 | 2.05 | 2.01 | 15.27 | 1.34 | 8.80 | 0.75 | 0.88 |
| | **22** | n.a. | n.a. | n.a. | 3.24 | 0.92 | 28.33 | 0.92 | 28.33 | 4.49 | 0.36 | 8.02 | 0.28 | 0.39 | 3.78 | 0.58 | 15.37 | 0.54 | 0.63 |
| | **23** | n.a. | n.a. | n.a. | 3.74 | 1.34 | 35.77 | 1.34 | 35.77 | 4.27 | 1.89 | 44.33 | 1.24 | 1.41 | 4.83 | 0.54 | 11.20 | 0.31 | 0.40 |
| | **24** | n.a. | n.a. | n.a. | 1.66 | 0.77 | 46.51 | 0.77 | 46.51 | 2.19 | 0.61 | 27.90 | 0.60 | 0.79 | 3.34 | 0.65 | 19.33 | 0.42 | 0.84 |
| | **25** | n.a. | n.a. | n.a. | 1.83 | 0.95 | 51.96 | 0.95 | 51.96 | 3.47 | 1.96 | 56.43 | 1.09 | 2.06 | 4.52 | 0.76 | 16.74 | 0.32 | 0.80 |
| **MDCK** | **1** | 1.62 | 0.25 | 15.50 | 1.55 | 0.24 | 15.75 | 0.25 | 15.75 | 0.93 | 0.18 | 19.14 | 1.22 | 0.71 | 0.85 | 0.26 | 30.39 | 1.93 | 1.03 |
| | **2** | 11.73 | 0.75 | 6.37 | 10.45 | 0.57 | 5.48 | 0.75 | 6.37 | 10.29 | 1.68 | 16.31 | 2.56 | 2.25 | 6.76 | 1.49 | 22.09 | **3.47** | 2.00 |
| | **3** | 6.45 | 0.40 | 6.14 | 5.66 | 0.03 | 0.59 | 0.40 | 6.14 | 3.42 | 0.47 | 13.83 | 2.25 | 1.19 | 3.57 | 0.30 | 8.44 | 1.38 | 0.76 |
| | **4** | 6.55 | 0.41 | 6.20 | 6.48 | 0.53 | 8.13 | 0.53 | 8.13 | 6.69 | 1.14 | 17.00 | 2.09 | 2.16 | 4.21 | 0.71 | 16.90 | 2.08 | 1.35 |
| | **6** | 9.24 | 0.40 | 4.37 | 9.56 | 0.57 | 6.00 | 0.57 | 6.00 | 12.13 | 1.83 | 15.13 | 2.52 | **3.20** | 14.64 | 2.90 | 19.79 | **3.30** | **5.05** |
| | **7** | 3.47 | 0.28 | 8.16 | 3.72 | 0.01 | 0.30 | 0.28 | 8.16 | 2.89 | 0.59 | 20.43 | 2.50 | 2.09 | 3.88 | 2.03 | 52.29 | **6.41** | **7.16** |
| | **8** | 8.21 | 0.34 | 4.16 | 8.40 | 0.03 | 0.33 | 0.34 | 4.16 | 6.75 | 0.26 | 3.89 | 0.94 | 0.77 | 5.13 | 0.37 | 7.15 | 1.72 | 1.07 |
| | **9** | 1.65 | 0.38 | 22.91 | 2.22 | 0.19 | 8.72 | 0.38 | 22.91 | 2.31 | 0.46 | 19.71 | 0.86 | 1.20 | 3.47 | 0.41 | 11.80 | 0.52 | 1.08 |
| | **10** | 9.84 | 0.61 | 6.24 | 9.89 | 0.10 | 1.03 | 0.61 | 6.24 | 12.21 | 0.92 | 7.56 | 1.21 | 1.50 | 13.02 | 2.80 | 21.53 | **3.45** | **4.57** |
| | **11** | 7.63 | 0.40 | 5.20 | 7.20 | 0.24 | 3.32 | 0.40 | 5.20 | 5.45 | 0.58 | 10.60 | 2.04 | 1.46 | 5.96 | 1.04 | 17.44 | **3.35** | 2.62 |
| | **12** | 6.92 | 0.53 | 7.65 | 7.04 | 0.09 | 1.27 | 0.53 | 7.65 | 13.02 | 1.45 | 11.16 | 1.46 | 2.74 | 10.74 | 1.24 | 11.57 | 1.51 | 2.35 |
| | **13** | 7.30 | 0.52 | 7.15 | 7.92 | 0.53 | 6.69 | 0.53 | 7.15 | 5.87 | 1.23 | 20.88 | 2.92 | 2.31 | 6.26 | 1.66 | 26.48 | **3.71** | **3.12** |
| | **14** | 1.36 | 0.32 | 23.64 | 1.56 | 0.06 | 3.79 | 0.32 | 23.64 | 3.08 | 0.99 | 32.01 | 1.35 | **3.07** | 3.86 | 2.05 | 53.22 | 2.25 | **6.38** |
| | **15** | 15.69 | 1.14 | 7.28 | 14.90 | 2.34 | 15.69 | 2.34 | 15.69 | 11.27 | 3.18 | 28.22 | 1.80 | 1.36 | 12.28 | 3.85 | 31.32 | 2.00 | 1.64 |
| | **16** | 2.33 | 0.51 | 21.69 | 3.46 | 0.82 | 23.69 | 0.82 | 23.69 | 3.68 | 1.32 | 35.73 | 1.51 | 1.61 | 5.36 | 1.14 | 21.26 | 0.90 | 1.39 |

* n = 10

°n = 4 (Vero); n = 2 (MDCK)

[#] n = 5 (Vero); n = 6 (MDCK)
